# A Study on Carbon Fiber Composites with Low-Melting-Point Polyester Nonwoven Fabric Reinforcement: A Highly Effective Electromagnetic Wave Shield Textile Material

**DOI:** 10.3390/polym14061181

**Published:** 2022-03-16

**Authors:** Jia-Horng Lin, Po-Wen Hsu, Chen-Hung Huang, Mei-Feng Lai, Bing-Chiuan Shiu, Ching-Wen Lou

**Affiliations:** 1 College of Material and Chemical Engineering, Minjiang University, Fuzhou 350108, China; jhlin@fcu.edu.tw (J.-H.L.); bcshiu@mju.edu.cn (B.-C.S.); 2 Advanced Medical Care and Protection Technology Research Center, Department of Fiber and Composite Materials, Feng Chia University, Taichung City 407102, Taiwan; j0928451554@gmail.com; 3 School of Chinese Medicine, China Medical University, Taichung City 404333, Taiwan; 4 Advanced Medical Care and Protection Technology Research Center, College of Textile and Clothing, Qingdao University, Qingdao 266071, China; 5 Department of Aerospace and Systems Engineering, Feng Chia University, Taichung City 40724, Taiwan; 6 Laboratory of Fiber Application and Manufacturing, Department of Fiber and Composite Materials, Feng Chia University, Taichung City 40724, Taiwan; 7 Department of Bioinformatics and Medical Engineering, Asia University, Taichung City 413305, Taiwan; 8 Department of Medical Research, China Medical University Hospital, China Medical University, Taichung City 404333, Taiwan; 9 Fujian Key Laboratory of Novel Functional Fibers and Materials, Minjiang University, Fuzhou 350108, China

**Keywords:** textile material, sandwich construction, needle-bonded, electromagnetic wave shield

## Abstract

In this study, a low-melting-point polyester nonwoven fabric (L), a nylon spacer fabric (N), and a carbon fiber woven fabric (C) are laminated in different orders and then needle-bonded at a depth of 15.0 cm to form NLC, NLN, CLC, and CLN composites with a sandwich construction. Regardless of the lamination order, four composite types exhibit high tensile strengths and tearing strengths. Based on the ASTM D4935-18 test standard, the electromagnetic wave shielding measurement is conducted in a frequency range of 1~3 GHz. The two groups—NLC and CLN—demonstrate different electromagnetic wave shields, which are −45~−65 dB for the former, and −60 dB for the latter. According to FTTS-FA-003, in the specified requirements of the test method for electromagnetic shielding textiles, the proposed composites achieve level III, which is the highest standard, and are thus qualified for use in the aviation, construction, and commerce fields.

## 1. Introduction

The matters of electromagnetic wave pollution concerns people as they become increasingly conscious of their health. As a result, highly effective electromagnetic wave shields have great potential for commercial usage [1,2,3]. Current shields have a light weight, small thickness, large width, and powerful functions, and there is a diverse range of electromagnetic shields [4,5,6]. In the meantime, researchers face many challenges when it comes to the practical use of these shields. For example, the human body and electronic equipment require the development of soft and convenient laminated electromagnetic interference (EMI) protective gear [7,8]. Suitable materials for EMI protective gear include metals, conductive polymers, electromagnetic materials, and carbon system materials [9,10,11,12]. To debilitate electromagnetic waves, materials with electrical conductivity apply a reflection mechanism, while materials with magnetic conductivity or a carbon system apply absorption dissipation. Despite their excellent electrical conductivity, metallic materials have the disadvantages of being high-cost, rigid, and easily oxidized and corroded, as well as having a high mass [13,14,15].

Zhu et al. (2021) altered the lamination sequence of nickel-plating nylon porous films, copper mesh, and carbon fibers, finding that the laminated composites exhibited different levels of mechanical properties, which was also the case when investigating the effectiveness of electromagnetic shielding. More specifically, the nylon/nickel film composites obtained an EMI shielding effectiveness (EMI SE) of −53.18 dB [16]. Similarly, Lai et al. (2021) employed a spinning ring in order to produce high-strength electrically conductive yarns that were then made into knitted fabrics and woven fabrics. The triple-layered woven fabrics, at a lamination angle of 0°/90°/45°, exhibited a maximal EMI SE of −45.96 dB [17]. Liang et al. (2021) proposed the use of carbon fiber felt composites, and investigated whether reinforcing the composites through lamination affected the EMI SE. When the layers were laminated at 90° instead of 0°, the EMI SE was reduced from −39.8 dB to −18.7 dB [18]. Moreover, Zhang et al. (2021) coated China grass in a Ti3AlC2 solution and a polycaprolactone in order to examine its influence over the EMI SE. It was successful as a flame retardant and the coating increased the EMI SE; the optimal EMI SE was −33.5 dB [19]. Finally, Choi et al. (2020) examined the EMI SE of PU foam composites which contained nickel-plated glass fabrics, which was −10 dB in a frequency range of 6.5–17.5 GHz [20]. To sum up, the composite materials used for electromagnetic wave shields are commonly made using three steps: combining them with metallic materials, laminating carbon fiber materials with PU foam, or spraying a mixture of carbon and metal ions over the composites. Therefore, this study aims to use an efficient needle-bonded process to produce low-melting-point polyester (LMPET) nonwoven fabrics, which will then be laminated with carbon fiber woven fabrics and nylon spacer fabrics in different orders. The layers will be bonded via the needle-bonding process and hot pressing, forming sandwich-structured composites. Finally, the physical properties of composites will be evaluated depending on the fabric type and lamination order.

## 2. Materials and Methods

### 2.1. Materials

Low-melting-point polyester (LMPET, Far Eastern New Century Co., Taipei, Taiwan) fibers with a fineness of 4D, a length of 51 mm, and a single fiber strength of 3.4 g/d that had a skin–core structure were used. The melting point was 110 °C for the skin and 265 °C for the core. Carbon fiber woven fabrics (Yurak International Trading Co., Ltd., Taichung City, Taiwan) with a warp density of 12.5 ends/inch were made from a 3K carbon fiber tow. The needles used in the needle-bonding process had a specification of 15 × 16 × 25 × 31/2 M332 G53017 and were purchased from GROZ-BECKER, Albstadt, Baden-Wuerttemberg, Germany.

### 2.2. Preparation

**Figure 1:**
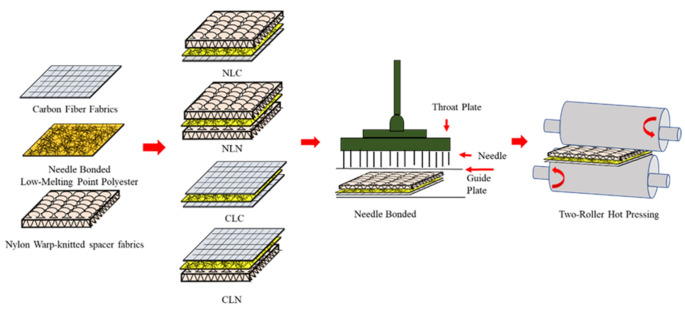
Preparation steps used for carbon fiber composites containing an LMPET nonwoven fabric as a reinforcement.

To start, LMPET fibers were made into nonwoven fabrics via the LMPET nonwoven fabric process at a needle-bonding depth of 1.25 cm. Next, nylon spacer fabrics were composed of interconnected loops that are produced using a warp-knitting frame (DH 1000-DNBAC, Dah Heer Industrial Co., Changhua, Taiwan). Afterwards, a needle-bonding system from Shoou Shyng Machinery (SUN-250SH, Chiefwell Engineering, New Taipei City, Taiwan) was employed to combine a LMPET fabric (abbreviated as L), a nylon spacer fabric (abbreviated as N), and a carbon fiber woven fabric (abbreviated as C), which were laminated in different orders, in order to form NLC, NLN, CLC, and NLC composites. The needle-bonding depth was 1.50 cm and the hot pressing temperature of the roller hot presser (Chiefwell Engineering, New Taipei City, Taiwan) was 130 °C. Figure 1 shows the illustrative diagram.

### 2.3. Testing

#### 2.3.1. Scanning Electron Microscopy (SEM)

A scanning electron microscope (S4800, HITACHI, Tokyo, Japan) was used to observe the thermal bonding morphology of the composites. Samples were fixed to the platform of the SEM using carbon fiber tape, and the surface morphology was observed at an operating voltage of 3 kV.

#### 2.3.2. Tensile Strength Test

The tensile strength and the elongation at fracture of samples are measured as speci-fied in the strip method of ASTM D 5035-11 with a computer universal testing machine (Hung Ta Instrument Co., Ltd., Taichung City, Taiwan). The distance between clamps is 7.5 cm; the test rate is 300 mm/min; and the samples have a size of 180 mm × 25.4 mm. Ten samples for each specification are taken along the machine direction (MD) and the cross machine direction (CD), respectively.

#### 2.3.3. Tearing Strength Test

As specified in ASTM D5035-06, the tearing strength of the composites was measured at a rate of 300 mm/min using a computer universal testing machine (Hung Ta Instrument Co., Ltd., Taichung City, Taiwan). Five samples (75 mm × 150 mm) from each specification were taken for the test. The sample cuttings had a depth of 15 mm and the distance between clamps was 25.4 mm.

#### 2.3.4. Bursting Strength Test

The bursting strength of samples is measured using a computer universal testing machine (Hung Ta Instrument Co., Ltd., Taichung City, Taiwan) as specified in ASTM D3787, Standard Test Method for Bursting Strength of Textiles—Constant-Rate-of-Traverse (CRT) Ball Burst Test. Samples have a size of 150 mm; the test rate is 100 mm/min; and a convex impact head is used.

#### 2.3.5. Electromagnetic Wave Shield

As specified in ASTM D4935-18, the electromagnetic wave shield of the composites was measured using a shielding effectiveness test sample holder (EM-2107A, Electro-Metrics Inc., Johnstown, NY, USA). A blank sample with the same thickness was tested as an electromagnetic wave shield so that it could be used as the reference for the rectification of the testing instrument that operates in a frequency range of 1–3 GHz. The shields mainly attenuate electromagnetic waves via three mechanisms, including reflection loss, absorption loss, and multiple reflection [21]. Usually, reflection loss is the main mechanism. For reflection to occur, the shields are required to have the carrier for r transmittable electric charges so that the interaction between the radiated electromagnetic field, electron, and holes can take place. Therefore, the shields are usually conductive. The second-most used mechanism is absorption loss. The shields need to be able to absorb electromagnetic waves, and thus, the shields need the magnetic dipole and the radiated electromagnetic field that interacts with the electromagnetic radiation. The last mechanism is multiple reflection, which means that each surface and interface of the shield can reflect the electromagnetic waves. The corresponding shields demand a greater surface area and pole interface. Whether the shields use absorption loss, reflection loss, or multiple reflection, the loss of electromagnetic waves was presented in dB [22,23].

## 3. Results and Discussion

### 3.1. SEM Analysis

**Figure 2:**
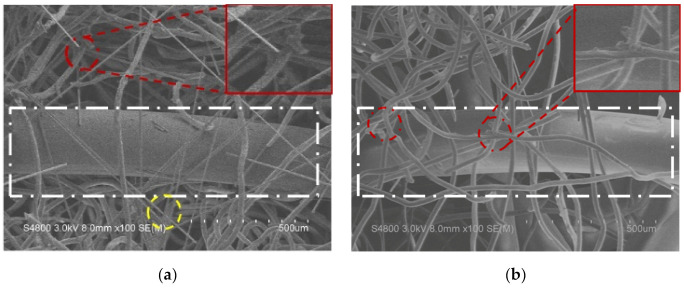

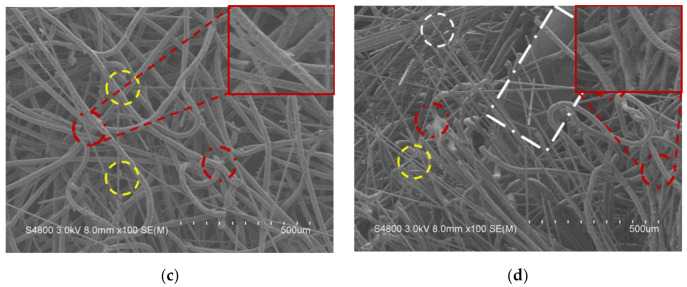
SEM images with a magnification of 8.0 mm × 100 SE (M) of (**a**) NLC, (**b**) NLN, (**c**) CLC, and (**d**) CLN composites observed at an operating voltage of 3.0 kV.

Figure 2 shows that the fibers of carbon fiber woven fabrics, nylon spacer fabrics, and LMPET nonwoven fabrics become firmly entangled due to the employment of the needle-bonding process [24,25]. The barbed needles efficiently moved and bonded the fibers with different layers, thereby mechanically strengthening the composites, which was deemed to be a typical result of the needle-bonded process for nonwoven fabrics [26,27,28,29]. To further improve the mechanical properties of the composites, a roller hot presser was used to melt the LMPET fibers, which, in turn, formed thermal bonding points [30,31,32]. Based on the literature [33], carbon fiber woven fabrics can withstand temperatures that exceed 1500 °C. The melting point is 235 °C for nylon, but low-melting-point polyester (LMPET) fibers are composed of a core–skin structure, where the melting point was 265 °C for the core and 110 °C for the skin. As the hot pressing temperature was 130 °C, the skin of the LMPET fibers was melted to form thermal bonding points. As indicated by the red circles in Figure 2a–d, the skin of the LMPET fibers was transformed into thermal bonding points that adhered to the other fibers, e.g., carbon fibers, as indicated by the yellow circles in Figure 2a,c,d, and nylon fibers, as indicated by the white rectangles in Figure 2a,b,d. Moreover, Figure 2a–d show that, regardless of the lamination order, all sample groups demonstrated thermal bonding via LMPET fibers as a result of the employment of hot pressing.

### 3.2. Tensile Strength

**Figure 3:**
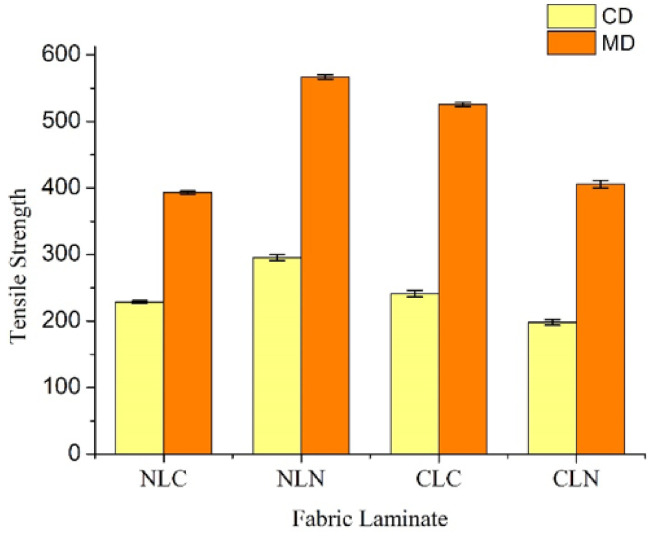
Tensile strengths of the NLC, NLN, CLC, and CLN composites.

Figure 3 and Table 1 show that the tensile strength along the machine direction (MD) is higher than that along the cross direction (CD), despite the lamination order of the composites. This result is consistent with the findings of a previous study [34]. In addition, Figure 3 demonstrates that the NLN group exhibited a greater tensile strength of 567.10 ± 3.35 N along the MD and of 295.46 ± 4.34 N along the CD. Nylon spacer fabrics (N) have a stiffer structure/pattern, with two layers of N on the top and bottom layers; NLN composites have a greater thickness of 5.49 and thus a greater tensile strength. The second group is composed of CLC composites that have a tensile strength along the MD of 525.63 ± 3.05 N, and a tensile strength along the CD of 241.35 ± 4.90 N. The needle-bonding process does not interfere with the surface of carbon fiber woven fabrics. The majority of damaged carbon fibers are due to the LMPET nonwoven layer becoming entangled with the LMPET fibers, which provides CLC composites with a thickness of 2.20 mm. The improvement in the tensile strength of thermal bonding points can be seen in Figure 1 and Figure 2. Based on the tensile strength data in Table 1, in addition to the difference in composition of the top and bottom layers, the thickness and the tensile strength of both the NLC group and the CLN group are also dependent on the needle-bonding process and hot pressing. The CLN group has a thickness of 3.56 mm, and the tensile strength along the MD is 405.70 ± 5.68, with that along the CD is 198.32 ± 4.38. The NLC group has a thickness of 4.83 mm, and the tensile strength along the MD is 393.26 ± 2.28 N, with that along the CD being 228.55 ± 1.96 N. Moreover, the NLC and CLN groups have the same density (0.17 g/cm^3^), while the NLN and CLC groups exhibit the lowest and the highest densities of 0.16 g/cm^3^ and 0.25 g/cm^3^ respectively. In addition, nylon spacer fabrics have a knitted pattern, and thus they resemble the elongation and elasticity of knits. NLC, NLN, and CLN groups share an elongation range of 30.10%~31.12%, and all composites (i.e., NLC, NLN, CLC, and CLN groups) have excellent tensile performance.

### 3.3. Tearing Strength

**Figure 4:**
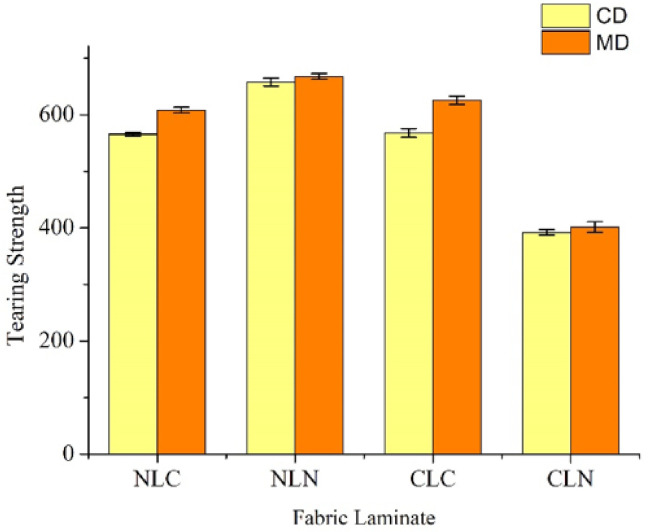
Tearing strengths of the NLC, NLN, CLC, and CLN composites.

Figure 4 and Table 2 show the tearing strengths along the MD and the CD of the composites. The tearing strengths along the MD are higher, which follows the same trend as the tensile strengths in Figure 3. After comparing the tearing strengths and the tensile strengths along the MD and the CD, the results agree with the findings of previous studies [35,36,37]. This also substantiates the claim that a different lamination order can withstand the maximal lateral damage, as the NLN group bears the maximal tearing strength along the CD, which is 658.07 ± 6.91 N, with that along the MD being 667.80 ± 5.14. Nylon spacer fabrics are composed of warp-knitted nylon filament loops. Significantly, as the interconnected loops are warped, they provide the nylon spacer fabrics with a dimensional structure and a greater tearing strength along the MD.

The CLC group is ranked in second place, and the tearing strength along the CD and the MD is 568.03 ± 7.70 N and 667.80 ± 5.14, respectively. Carbon fiber woven fabrics exhibit a higher tearing strength because LMPET fibers and hot pressing provide thermal bonding points. Moreover, the NLC and CLN groups show different tearing strengths with the same lamination layers, but in a different lamination order. The NLC group has tearing strengths along the CD and the MD of 565.50 ± 3.22 N and 608.53 ± 5.07 N, respectively, while the CLN group has a tearing strength along the CD and the MD of 392.30 ± 4.64 N and 401.52 ± 9.60 N, respectively. Compared to the CLN group, the NLC group has a nylon spacer fabric as the top layer, an LMPET nonwoven fabric as the interlayer, and a carbon fiber woven fabric as the bottom layer. When the NLC group undergoes the needle-bonding process, carbon fibers and LMPET fibers become entangled with the nylon fibers, which, in turn, makes the carbon fibers exhibit a better thermal bonding effect during the hot pressing process.

The CLN group is composed of a carbon fiber woven fabric top layer, an LMPET nonwoven fabric interlayer, and a nylon spacer fabric bottom layer. The carbon fibers and LMPET fibers undergo substantial bonding to nylon loops during the needle-bonding process, after which the CLN group exhibits a fluffy surface. The thermal bonding points usually become visible at the surface after hot pressing, so the CLN group shows a comparatively lower tearing strength than the NLC group, as shown in Figure 1. Table 2 indicates that the NLC, NLN, and CLN groups contain nylon spacer fabrics that have a knitting structure, and that the elongation is between 33.61% and 37.51%. By contrast, the CLC group (without nylon spacer fabric) exhibits an elongation of 17.21~18.21%.

### 3.4. Bursting Strength

**Figure 5:**
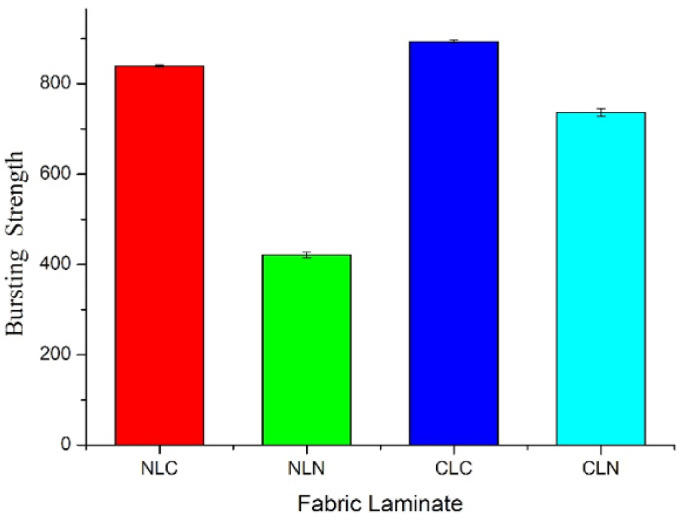
Bursting strengths of the NLC, NLN, CLC, and CLN composites.

Figure 5 and Table 3 show that with a different lamination order, combined with the needle-bonding process and hot pressing, the CLC group (w/o a nylon spacer fabric) is able to exhibit the highest bursting strength, which is 893.73 ± 3.33 N. The LMPET interlayer is firmly bonded with the top/bottom layers of the carbon fiber woven fabrics, providing the CLC group with the lowest thickness. Subsequently, the area of the thermal bonding points of the CLC group increases, resulting in the maximal bursting strength.

The NLC group has a bursting strength of 839.85 ± 2.36 N, which is ranked in second place. The bottom layer (i.e., the carbon fiber woven fabric) encounters the lowest level of damage, and then the carbon fibers are thermally bonded with the LMPET fibers, causing the second-highest bursting strength. Moreover, the CLN group uses needle-bonding on the carbon fibers (top layer) and LMPET fibers (interlayer), and they are also damaged, which firmly bonds them to the nylon spacer fabric. After hot pressing, the CLN group tends to be softer and exhibits a bursting strength of 736.96 ± 8.60 N. Finally, the NLN group exhibits the lowest bursting strength of 420.65 ± 5.94 N, which is ascribed to the top and bottom layers being nylon spacer fabrics. Nylon spacer fabrics are formed of interconnected loops, and they appear to be sleek, which then compromises the bursting strength of the NLN group [38,39,40,41].

### 3.5. Electromagnetic Wave Shield

**Figure 6:**
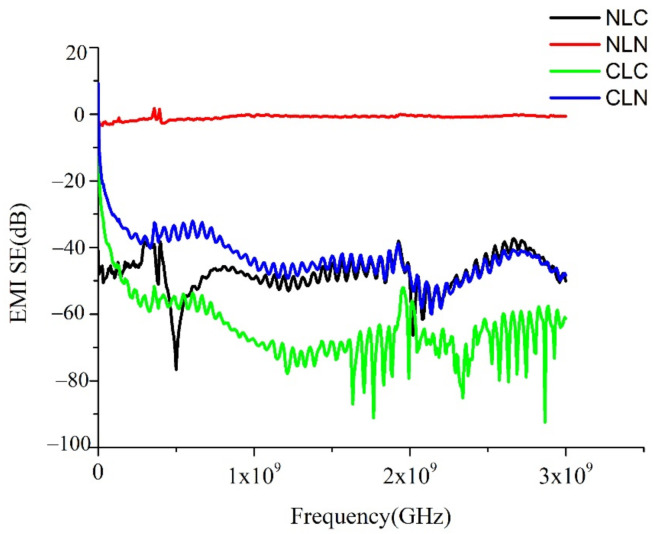
s of the NLC, NLN, CLC, and CLN composites.

Figure 6 shows the electromagnetic wave shields of the NLC, NLN, CLC, and CLN composites. Civil textiles with electromagnetic wave shields can be ranked as −30 dB for level I, −20 dB for level II, and −10 dB for level III [17,42,43,44]. From the perspective of lamination order, the needle-bonded, sandwich-structured composites show electromagnetic wave shields at 1~3 GHz that can be ranked from highest to lowest in terms of the NLC, CLC and CLN groups. More specifically, the CLC group exhibits the maximal electromagnetic wave shield at 1.7~2 GHz as 91.11 dB. The NLC group has an electromagnetic wave shield of −45~−65 dB at 1~3 GHz, while the CLN group has an electromagnetic wave shield of −60.02 dB at 2 GHz. The constituent carbon fiber woven fabrics provide the NLC, CLC, and CLN groups with an electromagnetic wave shield; furthermore, the needle-bonding process firmly bonds the fibers together, which then contributes to an excellent conductive network that attenuates electromagnetic waves via the three mechanisms of absorption loss, reflection loss, and multiple reflection [45]. With the needle-bonding process, carbon fiber woven fabrics attain an excellent shielding efficacy, especially the CLC group, as it contains two layers of carbon fiber woven fabrics. The CLC group exhibits the maximal electromagnetic wave shield. The more shielding layers, the greater the shielding efficacy, which is in keeping with the findings of other studies [46]. By contrast, the NLN group cannot shield electromagnetic waves because it does not contain carbon fiber woven fabrics. The test results suggest that all of the sandwich-structured composites meet the test standard, and that they have a flexible range that can be applied to block electromagnetic waves at different frequencies in different fields, such as the aviation, construction, and commerce fields.

## 4. Conclusions

In this study, the performance of the needle-bonding process and hot pressing demonstrates that both can successfully form carbon fiber composites with LMPET nonwoven fabric reinforcement. The manufacturing process is proven to be efficient and simple. The SEM observation indicates that the NLC and CLN groups demonstrate distinct thermal bonding points that can mechanically improve carbon fiber woven fabric and nylon spacer fabric in terms of the tensile strength, tearing strength, and bursting strength. Additionally, the sandwich-structured composites demonstrate that they are excellent electromagnetic wave shields that exceed the electromagnetic wave shield textile standard. The test results indicate that the proposed products, which are composed of needle-bonded composites, attain the electromagnetic wave shield and mechanical properties that are required by the aviation, construction, and commerce fields.

## Figures and Tables

**Table 1:** Tensile strengths of sandwich-structured composites.

Sample	Direction	Tensile Strength (N)	Elongation (%)	Thickness (mm)	Density (g/cm^3^)
NLC	CD	228.55 ± 1.96	30.10	4.83	0.17
	MD	393.26 ± 2.28	31.10	4.83	0.17
NLN	CD	295.46 ± 4.34	30.09	5.49	0.16
	MD	567.10 ± 3.35	30.01	5.49	0.16
CLC	CD	241.35 ± 4.90	8.90	2.20	0.25
	MD	525.63 ± 3.05	10.01	2.20	0.25
CLN	CD	198.32 ± 4.38	30.15	3.56	0.17
	MD	405.70 ± 5.68	31.12	3.56	0.17

Note. MD is the machine direction and CD is the cross machine direction.

**Table 2:** Tearing strengths.

Sample	Direction	Tensile Strength (N)	Elongation (%)	Thickness (mm)
NLC	CD	565.50 ± 3.22	37.41	4.83
	MD	608.53 ± 5.07	33.61	4.83
NLN	CD	658.07 ± 6.91	37.81	5.49
	MD	667.80 ± 5.14	37.51	5.49
CLC	CD	568.03 ± 7.70	17.21	2.20
	MD	625.75 ± 7.25	18.21	2.20
CLN	CD	392.30 ± 4.64	34.81	3.56
	MD	401.52 ± 9.60	35.51	3.56

**Table 3:** Bursting strengths.

Sample	Bursting Strength (N)	Thickness (mm)
NLC	839.85 ± 2.36	4.83
NLN	420.65 ± 5.94	5.49
CLC	893.73 ± 3.33	2.20
CLN	736.96 ± 8.60	3.56

## Data Availability

All data relevant to the study are included in the article.

## References

[1] Kim B.-J., Kim K.-W. (2020). Carbon fiber-reinforced composites for EMI shielding. Materials for Potential EMI Shielding Applications.

[2] Vijay S., Tugirumubano A., Go S.H., Kwac L.K., Kim H.G. (2020). Carbon fiber-reinforced polymer-metal wire mesh hybrid composite for EMI shielding. Materials for Potential EMI Shielding Applications.

[3] Chung D.D. (2020). Materials for electromagnetic interference shielding. Mater. Chem. Phys..

[4] Jia L.-C., Xu L., Ren F., Ren P.-G., Yan D.-X., Li Z.-M. (2019). Stretchable and durable conductive fabric for ultrahigh performance electromagnetic interference shielding. Carbon.

[5] Tan Y.-J., Li J., Gao Y., Li J., Guo S., Wang M. (2018). A facile approach to fabricating silver-coated cotton fiber non-woven fabrics for ultrahigh electromagnetic interference shielding. Appl. Surf. Sci..

[6] Ravindren R., Mondal S., Nath K., Das N.C. (2019). Prediction of electrical conductivity, double percolation limit and electromagnetic interference shielding effectiveness of copper nanowire filled flexible polymer blend nanocomposites. Compos. Part B Eng..

[7] Yuan Y., Yin W., Yang M., Xu F., Zhao X., Li J., Peng Q., He X., Du S., Li Y. (2018). Lightweight, flexible and strong core-shell non-woven fabrics covered by reduced graphene oxide for high-performance electromagnetic interference shielding. Carbon.

[8] Yan R., Zhang Q., Shi B., Qin Z., Wei S., Jia L. (2020). Investigating the integral-structure of HRBP/CHP/CF consisting of non-woven flexible inter/intra-ply hybrid composites: Compression, puncture-resistance, electromagnetic interference shielding effectiveness. Compos. Struct..

[9] Luo X., Chung D. (1999). Electromagnetic interference shielding using continuous carbon-fiber carbon-matrix and polymer-matrix composites. Compos. Part B Eng..

[10] Chung D. (2001). Electromagnetic interference shielding effectiveness of carbon materials. Carbon.

[11] Moučka R., Sedlačík M., Prokeš J., Kasparyan H., Valtera S., Kopecký D. (2020). Electromagnetic interference shielding of polypyrrole nanostructures. Synth. Met..

[12] Park J.-B., Rho H., Cha A.-N., Bae H., Lee S.H., Ryu S.-W., Jeong T., Ha J.-S. (2020). Transparent carbon nanotube web structures with Ni-Pd nanoparticles for electromagnetic interference (EMI) shielding of advanced display devices. Appl. Surf. Sci..

[13] Ren W., Zhu H., Yang Y., Chen Y., Duan H., Zhao G., Liu Y. (2020). Flexible and robust silver coated non-woven fabric reinforced waterborne polyurethane films for ultra-efficient electromagnetic shielding. Compos. Part B Eng..

[14] Chen L., Guo K., Zeng S.-L., Xu L., Xing C.-Y., Zhang S., Li B.-J. (2020). Cross-stacking aligned non-woven fabrics with automatic self-healing properties for electromagnetic interference shielding. Carbon.

[15] Li M.-Z., Jia L.-C., Zhang X.-P., Yan D.-X., Zhang Q.-C., Li Z.-M. (2018). Robust carbon nanotube foam for efficient electromagnetic interference shielding and microwave absorption. J. Colloid Interface Sci..

[16] Zhu H., Fu K., Yang B., Li Y. (2021). Nickel-coated nylon sandwich film for combination of lightning strike protection and electromagnetic interference shielding of CFRP composite. Compos. Sci. Technol..

[17] Lai M.-F., Lou C.-W., Lin T.A., Wang C.-H., Lin J.-H. (2021). High-strength conductive yarns and fabrics: Mechanical properties, electromagnetic interference shielding effectiveness, and manufacturing techniques. J. Text. Inst..

[18] Liang J., Bai M., Gu Y., Wang S., Li M., Zhang Z. (2021). Enhanced electromagnetic shielding property and anisotropic shielding behavior of corrugated carbon fiber felt composite and its sandwich structure. Compos. Part A Appl. Sci. Manuf..

[19] Cheng W., Zhang Y., Tao Y., Lu J., Liu J., Wang B., Song L., Jie G., Hu Y. (2021). Durable Electromagnetic Interference (EMI) Shielding Ramie Fabric with Excellent Flame Retardancy and Self-healing performance. J. Colloid Interface Sci..

[20] Choi W.-H., Kwak B.-S., Kweon J.-H., Nam Y.-W. (2020). Radar-absorbing foam-based sandwich composite with electroless nickel-plated glass fabric. Compos. Struct..

[21] Geetha S., Satheesh Kumar K., Rao C.R., Vijayan M., Trivedi D. (2009). EMI shielding: Methods and materials—A review. J. Appl. Polym. Sci..

[22] Liu P., Yao Z., Zhou J., Yang Z., Kong L.B. (2016). Small magnetic Co-doped NiZn ferrite/graphene nanocomposites and their dual-region microwave absorption performance. J. Mater. Chem. C.

[23] Liu P., Ng V.M.H., Yao Z., Zhou J., Lei Y., Yang Z., Lv H., Kong L.B. (2017). Facile synthesis and hierarchical assembly of flowerlike NiO structures with enhanced dielectric and microwave absorption properties. ACS Appl. Mater. Interfaces.

[24] Zhao D., Mao K., Yang Y., Hamada H. (2017). Flexural behavior evaluation of needle-punched glass/jute hybrid mat reinforced polymer composites. Procedia Eng..

[25] El Wazna M., El Fatihi M., El Bouari A., Cherkaoui O. (2017). Thermo physical characterization of sustainable insulation materials made from textile waste. J. Build. Eng..

[26] Zhang Y., Li T.-T., Shiu B.-C., Sun F., Ren H.-T., Zhang X., Lou C.-W., Lin J.-H. (2021). Eco-friendly versatile protective polyurethane/triclosan coated polylactic acid nonwovens for medical covers application. J. Clean. Prod..

[27] Das B., Debnath C., Ray P. (1987). Scanning electron microscope observations of nonwoven fabrics from jute and other textiles. Text. Res. J..

[28] Tian N., Wu S., Han G., Zhang Y., Li Q., Dong T. (2022). Biomass-derived oriented neurovascular network-like superhydrophobic aerogel as robust and recyclable oil droplets captor for versatile oil/water separation. J. Hazard. Mater..

[29] Dong T., Li Q., Tian N., Zhao H., Zhang Y., Han G. (2021). Concus Finn Capillary driven fast viscous oil-spills removal by superhydrophobic cruciate polyester fibers. J. Hazard. Mater..

[30] Wang X., Gong R., Dong Z., Porat I. (2007). Abrasion resistance of thermally bonded 3D nonwoven fabrics. Wear.

[31] Choi S., Lim J.H., Kim H.S. (2018). Effect of Bonding Temperature on the Adhesion Characteristics and Mechanical Properties of Non-woven LMPET/PET. Text. Sci. Eng..

[32] Fedorova N., Verenich S., Pourdeyhimi B. (2007). Strength optimization of thermally bonded spunbond nonwovens. J. Eng. Fibers Fabr..

[33] Lin M.-C., Lou C.-W., Lin J.-Y., Lin T.A., Lin J.-H. (2019). Mechanical property evaluations of flexible laminated composites reinforced by high-performance Kevlar filaments: Tensile strength, peel load, and static puncture resistance. Compos. Part B Eng..

[34] Anuar N.I.S., Zakaria S., Gan S., Chia C.H., Wang C., Harun J. (2019). Comparison of the morphological and mechanical properties of oil Palm EFB fibres and kenaf fibres in nonwoven reinforced composites. Ind. Crops Prod..

[35] Singh N., Rani A. (2014). Needle punched non woven of Sesbania aculeata (dhaincha) fiber. Int. J. Text. Fash. Technol..

[36] Lin T.R., Lin T.A., Lin M.-C., Lin Y.-Y., Lou C.-W., Lin J.-H. (2020). Using recycled high-strength polyester and Kevlar^®^ wastes to reinforce sandwich-structured nonwoven fabric: Structural effect and property evaluation. J. Clean. Prod..

[37] Huang C.H., Li T.T., Chuang Y.C., Lou C.W., Chen J.M., Lin J.H. (2012). The primary study on polyester/polypropylene sound-absorption nonwoven fabric. Adv. Mater. Res..

[38] Villalpando A., Easson M., Cheng H., Condon B. (2019). Use of cottonseed protein as a strength additive for nonwoven cotton. Text. Res. J..

[39] Chauhan V.K., Debnath S., Singh B. (2021). Optimizing bursting behavior of calendered needle-punched polyester fabrics. J. Text. Inst..

[40] Thangadurai K., Thilagavathi G., Bhattacharyya A. (2014). Characterization of needle-punched nonwoven fabrics for industrial air filter application. J. Text. Inst..

[41] Chang Y., Ma P., Jiang G. (2017). Energy absorption property of warp-knitted spacer fabrics with negative Possion’s ratio under low velocity impact. Compos. Struct..

[42] Ozen M.S., Sancak E. (2016). Investigation of electromagnetic shielding effectiveness of needle punched nonwoven fabrics with staple polypropylene and carbon fibres. J. Text. Inst..

[43] Lu L., Xing D., Teh K.S., Liu H., Xie Y., Liu X., Tang Y. (2017). Structural effects in a composite nonwoven fabric on EMI shielding. Mater. Des..

[44] Sancak E., Akalin M., Usta İ., Yuksek M., Özen M. (2018). The effects of fabric and conductive wire properties on electromagnetic shielding effectiveness and surface resistivity of interlock knitted fabrics. Fibers Polym..

[45] Liu P., Yao Z., Ng V.M.H., Zhou J., Kong L.B., Yue K. (2018). Facile synthesis of ultrasmall Fe_3_O_4_ nanoparticles on MXenes for high microwave absorption performance. Compos. Part A Appl. Sci. Manuf..

[46] Xia C., Yu J., Shi S.Q., Qiu Y., Cai L., Wu H.F., Ren H., Nie X., Zhang H. (2017). Natural fiber and aluminum sheet hybrid composites for high electromagnetic interference shielding performance. Compos. Part B Eng..

